# Nitras Argenti to the Uterine Cavity

**Published:** 1873-05

**Authors:** 


Nitras Argenti to the Uterine Cavity.—A simple and efficient means of applying nitrate of silver to the cavity of the uterus consists in the cloth tent, several times dipped in a saturated solution of the nitrate; and when dried it will be found a solid, semi-flexible stick of nitrate of silver, easily introduced into the uterine cavity, and retained for any desirable length of time. The tent may be thus medicated entire or only in part, according to the extent of surface it is desired to cauterize. Nitrate of silver applied in this way does not cause the violent pains which sometimes follow the application of the solid stick to the cavity as made in the ordinary way.

